# Primary Middle Mediastinal Hydatid Cyst in Pediatric Patients: A Case Report and Literature Review

**DOI:** 10.7759/cureus.85665

**Published:** 2025-06-09

**Authors:** Zheer Ako, Salam A Bermani, Aram Baram

**Affiliations:** 1 Cardiothoracic and Vascular Surgery, University of Sulaimani, Sulaymaniyah, IRQ; 2 Surgery, Faruk Medical City, Sulaymaniyah, IRQ; 3 Thoracic and Cardiovascular Surgery, College of Medicine, University of Sulaimani, Sulaymaniyah, IRQ

**Keywords:** differential diagnosis, mediastinal hydatid cyst, outcome measure, pediatrics, surgery

## Abstract

Hydatid disease is a parasitic infection caused by Echinococcus species. Mediastinal involvement is rare, especially in children. We report a case of a four-year and eight-month-old girl who presented with progressive shortness of breath, cyanosis, and weight loss over two months. Imaging revealed a large cystic lesion in the middle mediastinum, compressing the trachea and superior vena cava. Surgical excision confirmed a hydatid cyst, and postoperatively, the patient received antihelmintic therapy with a favorable recovery. Mediastinal hydatid cysts are rare but should be considered in endemic areas. Early diagnosis and surgical intervention are crucial for optimal outcomes.

## Introduction

Hydatid disease is a zoonotic parasitic infection caused by the larval stage of *Echinococcus granulosus.* It is endemic in certain regions, notably the Middle East, Far East, and South America, particularly in rural areas where close contact with carnivores, sheep, and cattle is common [1,2]. Humans serve as accidental intermediate hosts, typically acquiring the infection through ingestion of water or food contaminated with eggs shed in the feces of definitive hosts. The disease predominantly affects the liver, lungs, and brain. In the pediatric population, pulmonary hydatid cysts are more prevalent than hepatic involvement [3].

Intrathoracic extrapulmonary hydatid cysts may develop in locations such as the pleural space, pleural fissures, chest wall, diaphragm, mediastinum, pericardium, and myocardium. Primary involvement of the mediastinum is exceedingly rare, accounting for less than 1% of all thoracic hydatid cysts, particularly as a primary site of infection. Unlike mucosal surfaces, the serosal lining of the mediastinum provides a conducive environment for cyst growth [4].

Review of the literature reveals a lack of consensus regarding the diagnosis and management of pediatric mediastinal hydatid cysts, with existing recommendations largely based on limited data. This report presents the case of a four-year-old girl with a primary middle mediastinal hydatid cyst, emphasizing the associated diagnostic and therapeutic challenges.

## Case presentation

A four-year and eight-month-old girl from a rural area presented with gradually worsening shortness of breath over several days. Her symptoms progressed, accompanied by coughing and episodes of cyanosis. She was unable to lie flat and required the use of a pillow at night for comfort. There was no history of fever or night sweats; however, her mother reported decreased appetite, generalized weakness, and weight loss. Initially, her pediatrician treated her symptoms as an upper respiratory tract infection over two months without improvement.

Diagnostic workup

**Figure 1:**
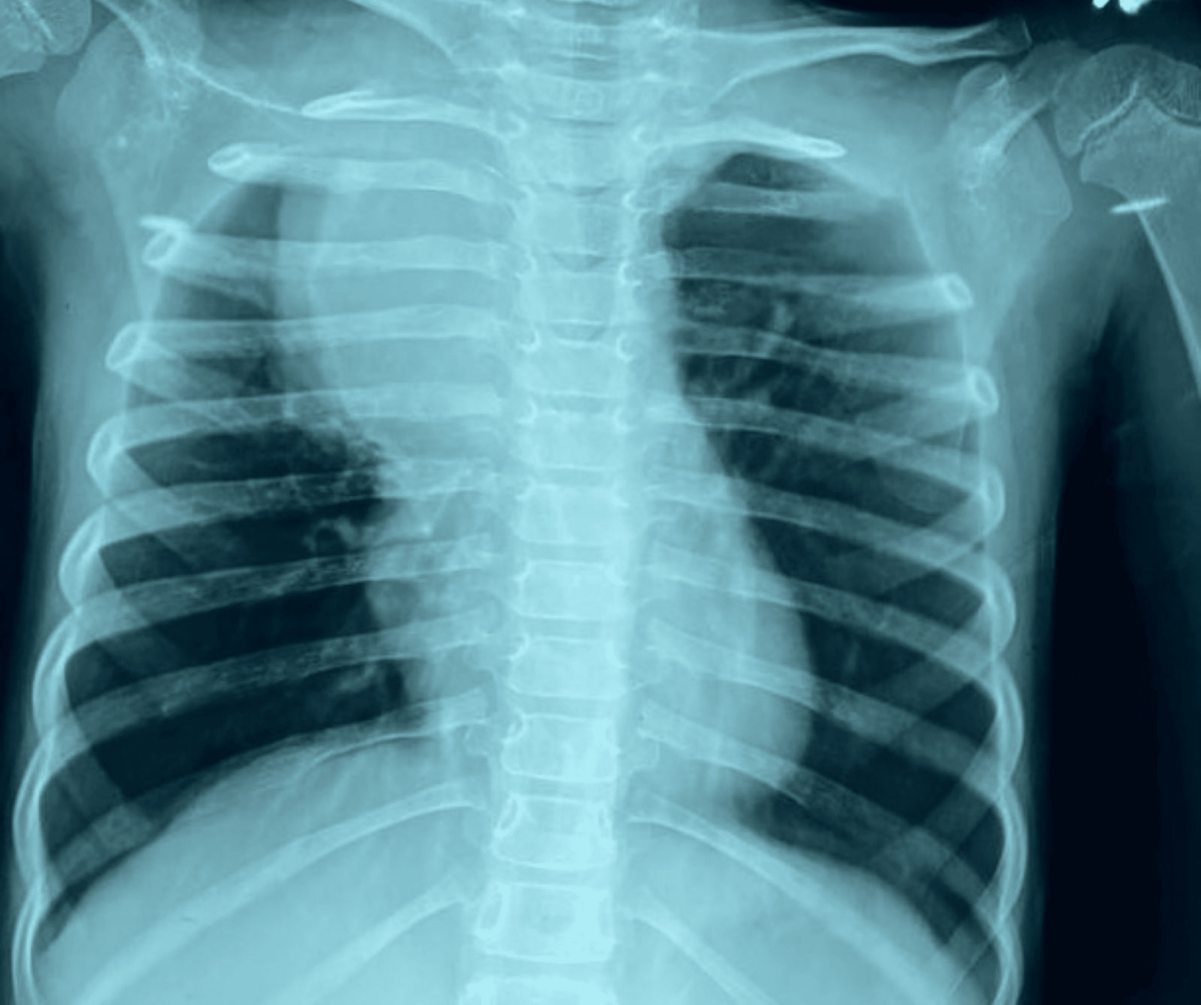
Posteroanterior chest radiograph showing mediastinal widening predominantly on the right side, with a well-defined, homogeneous opacity exerting mass effect on the upper lobe of the right lung.

**Figure 2:**
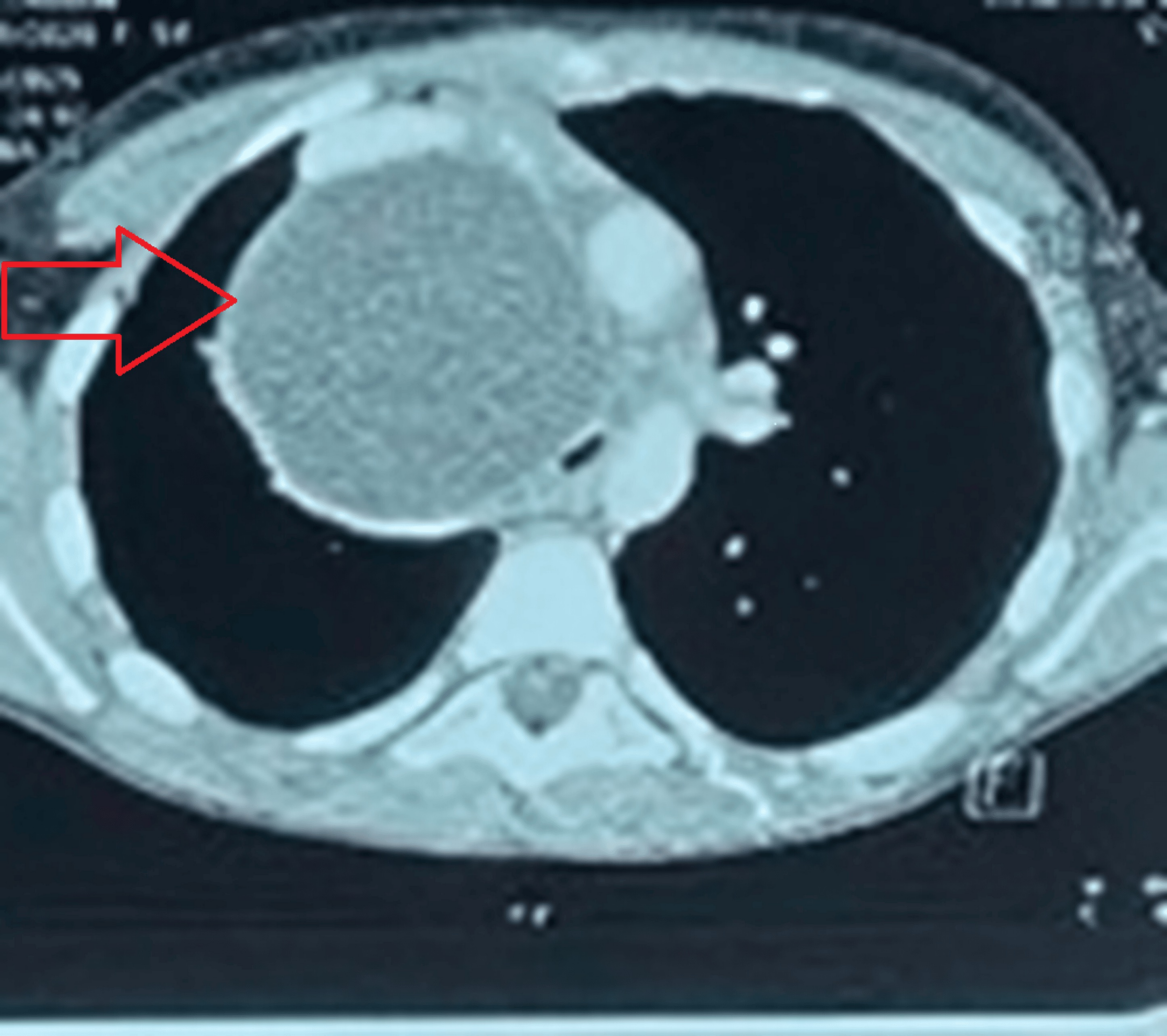
Chest computed tomography scan showing an isolated cystic lesion in the middle mediastinum (red arrow), compressing the trachea and the superior vena cava.

Initial chest radiography demonstrated right-sided mediastinal widening characterized by a well-defined, homogeneous, round opacity (Figure 1). Given these findings, a contrast-enhanced computed tomography (CT) scan of the chest was performed, which revealed a large, cystic lesion located in the middle mediastinum (Figure 2). The lesion was causing significant compression of the trachea, superior vena cava (SVC), and right upper lobe of the lung.

Treatment and surgical management

**Figure 3:**
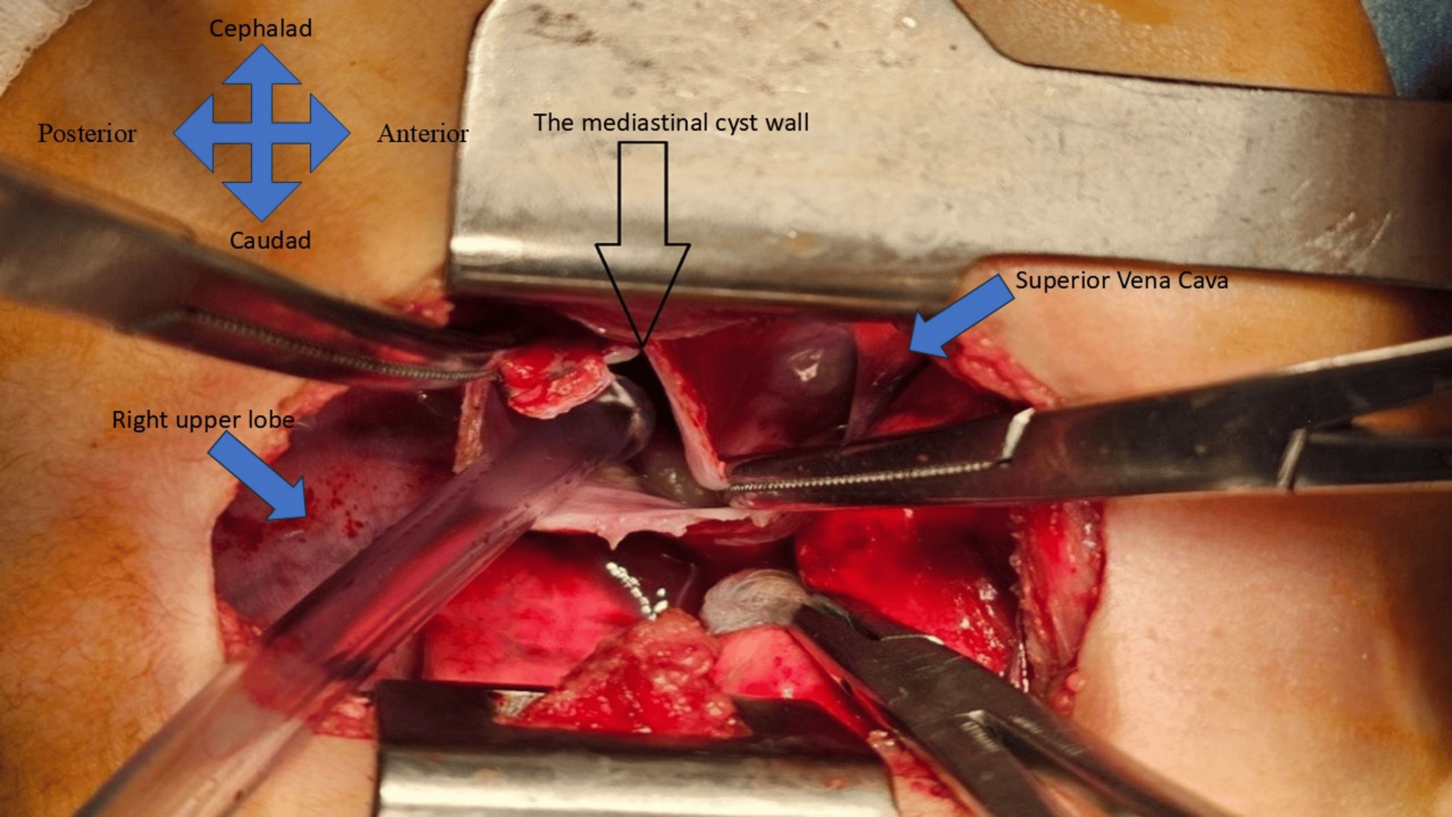
Intraoperative findings showing the cyst located behind the superior vena cava. The black arrow indicates the cyst.

Following stabilization of the patient’s general condition, the surgical team opted for a muscle-sparing right posterolateral thoracotomy (Figure 3). Intraoperative findings revealed a sizable (10 x 10 cm) cystic mass in the mid-mediastinum. The lesion was anatomically located lateral to the trachea, with the SVC anteriorly and the azygos vein inferiorly. Once the lesion was completely isolated from the surrounding structures using povidone-iodine-soaked packs, the cyst aspiration yielded a clear fluid. Upon opening the lesion, an endocyst of a hydatid cyst was visible. Although the germinal layer is the only living part of the parasite, surgeons typically remove the laminated membrane as well. The laminated membrane, which lies adjacent to the germinal layer, is identifiable as a thick, white membrane. Removal of this membrane is important to ensure thorough clearance of the cyst contents and reduce the risk of recurrence. The procedure was completed without complications. The patient was started on albendazole therapy (10-15 mg/kg/day BID) postoperatively and was discharged in stable condition on the second postoperative day.

## Discussion

The term *hydatid cyst* was first introduced by Rudolphi in 1800 to describe the pathological condition caused by the larval stage of *Echinococcus granulosus*. In this zoonotic infection, the adult tapeworm inhabits the jejunum of definitive hosts, typically dogs. Eggs released in the feces are ingested by intermediate hosts, such as sheep, cattle, or humans, through contaminated water or food. Once ingested, the eggs hatch in the gastrointestinal tract, releasing oncospheres that penetrate the intestinal wall and enter the portal circulation. The liver serves as the primary filter, either eliminating most larvae or transforming them into hydatid cysts. However, some larvae may bypass the hepatic filter, reaching the right heart and subsequently the pulmonary circulation secondary filter, before potentially disseminating to other organs [4,5].

Mediastinal cysts make up about 25% of all masses in the mediastinum, with common types being bronchogenic cysts, thymic cysts, pericardial cysts, and lymphangiomas. Primary mediastinal hydatid cysts, particularly those with no pleuropulmonary involvement, are exceedingly rare. In a large series of over 23,000 hydatid disease cases, Eroğlu et al. reported mediastinal involvement in only 0.1% of patients [3]. Similarly, Id el haj et al. observed a 0.5% incidence among 1,619 cases [6].

Primary mediastinal hydatid cysts predominantly affect adults. In a retrospective analysis of 254 patients with thoracic hydatidosis over a decade, Semerkant and Esme identified only four cases (1.6%) of mediastinal involvement, with patient ages ranging from 47 to 74 years (mean: 59 years) [1]. Other studies, such as those by Eroğlu et al. (427 cases) [3] and Tulay (158 cases) [2], reported median patient ages of 28.4 and 33.7 years, respectively. In contrast, our patient, a four-year and eight-month-old girl, represents an exceptionally rare case of primary mediastinal hydatid disease in the pediatric population.

The clinical presentation of hydatid cysts is variable and often depends on the cyst's size, location, and the degree of compression of adjacent structures. Common symptoms include retrosternal chest pain, cough, dysphagia, and dyspnea, especially if the cyst compresses the trachea or SVC. In our case, the child presented with stridor and dyspnea, likely secondary to tracheal compression, carinal, and bronchial compression [7,8].

Diagnosis is primarily based on clinical suspicion, especially in endemic regions, supported by radiological imaging. Chest radiography, CT, and magnetic resonance imaging (MRI) are essential for identifying cyst characteristics and their anatomical relationships. CT is particularly useful in delineating the size, density, and compressive effects of the lesion.

While serological tests, such as indirect hemagglutination assay (IHA) and enzyme-linked immunosorbent assay (ELISA), can support the diagnosis of hydatid disease by detecting antibodies against Echinococcus antigens, their sensitivity is variable and often limited. In particular, these tests frequently produce false-negative results when the cysts remain intact and are well encapsulated, as the immune system’s exposure to parasitic antigens is minimal in such cases. Consequently, negative serology does not exclude hydatid disease, especially in early or localized infections, and diagnosis often relies heavily on clinical presentation and imaging findings [7].

Fine-needle aspiration is contraindicated due to the risk of cyst rupture and anaphylaxis. As in our case, definitive diagnosis is frequently made intraoperatively. Our patient underwent chest X-ray and CT imaging; serological testing was not performed. Abdominal ultrasonography ruled out hepatic involvement. Differential diagnoses considered included foregut duplication cysts (e.g., bronchogenic and esophageal duplication cysts), neuroenteric cysts, meningoceles, thymic cysts, and pericardial cysts. Unlike hydatid cysts, these lesions typically do not display internal septations, multiplicity, or calcifications, nor do they exhibit significant wall enhancement on imaging [8]. Based on imaging and clinical context, hydatid cyst and bronchogenic cyst were the leading differentials.

Complications of mediastinal hydatid cysts may include rupture, fistula formation, secondary infection, and compression of vital structures. Surgical intervention remains the definitive treatment. The objective of surgery is to remove the cyst contents-particularly the germinal and laminated membranes-while minimizing the risk of spillage and recurrence. Total pericystectomy is preferred when anatomically feasible; however, partial pericystectomy is commonly performed when the cyst abuts critical structures.

Surgical approaches for mediastinal hydatid cysts are tailored according to the cyst’s location, size, and relation to surrounding vital structures. Common techniques include minimally invasive methods such as video-assisted thoracic surgery (VATS), which may be suitable for smaller, well-accessible cysts, as well as open procedures like posterolateral or anterolateral thoracotomy and median sternotomy, which offer better exposure for larger or more complex lesions [6-8]. In our case, a posterolateral thoracotomy was chosen to provide optimal access to the middle mediastinum. However, complete cyst excision was contraindicated due to the cyst’s close adherence to critical vascular structures, including the SVC and azygos vein, as well as the trachea. To minimize the risk of injury, the surgical team performed a careful removal of the germinal membrane combined with a partial pericystectomy, effectively decompressing the cyst while preserving surrounding anatomy. This approach balances adequate disease clearance with patient safety and is often necessary when total resection risks significant morbidity.

The intraoperative use of scolicidal agents such as hypertonic saline, povidone-iodine, hydrogen peroxide, or chlorhexidine is aimed at sterilizing the cyst contents and minimizing the risk of dissemination and recurrence. However, their use remains controversial, particularly in delicate anatomical locations like the mediastinum, where spillage may be difficult to control and surrounding vital structures (e.g., major vessels and airway) are at risk of chemical injury [6-8]. While some guidelines advocate instilling a scolicidal agent into the cyst cavity before aspiration or membrane removal, others suggest that careful evacuation of cyst contents and protection of the operative field with gauze soaked in antiseptic (e.g., povidone-iodine) may suffice [5-8]. In pediatric patients, or when the cyst is adjacent to sensitive tissues, many surgeons opt to omit direct instillation to avoid potential complications. Current literature lacks consensus, and there is a need for further studies to establish standardized protocols, particularly in rare sites such as the mediastinum [8].

The use of albendazole as adjuvant therapy remains somewhat controversial. However, postoperative administration is generally recommended, particularly in cases involving cyst rupture or suspected dissemination [6-8]. Although there was no intraoperative evidence of rupture in our patient, albendazole was initiated prophylactically to reduce the risk of recurrence and systemic dissemination.

## Conclusions

Primary mediastinal hydatid cysts in children are rare and can show various symptoms, posing a significant risk of serious problems. Quick diagnosis and proper surgery are essential for good results and reducing the chances of negative effects. Prompt diagnosis combined with timely and appropriate surgical intervention is critical to achieving favorable outcomes and minimizing the likelihood of adverse sequelae.
